# Effect of Dietary Starch Source and Concentration on Equine Fecal Microbiota

**DOI:** 10.1371/journal.pone.0154037

**Published:** 2016-04-29

**Authors:** Brittany E. Harlow, Laurie M. Lawrence, Susan H. Hayes, Andrea Crum, Michael D. Flythe

**Affiliations:** 1 Department of Animal and Food Sciences, University of Kentucky, Lexington, KY, 40546, United States of America; 2 Forage Animal Production Research Unit, Agricultural Research Service, United States Department of Agriculture, Lexington, KY, 40546, United States of America; Max Rubner-Institut, GERMANY

## Abstract

Starch from corn is less susceptible to equine small intestinal digestion than starch from oats, and starch that reaches the hindgut can be utilized by the microbiota. The objective of the current study was to examine the effects of starch source on equine fecal microbiota. Thirty horses were assigned to treatments: control (hay only), HC (high corn), HO (high oats), LC (low corn), LO (low oats), and LW (low pelleted wheat middlings). Horses received an all-forage diet (2 wk; d -14 to d -1) before the treatment diets (2 wk; d 1 to 14). Starch was introduced gradually so that horses received 50% of the assigned starch amount (high = 2 g starch/kg BW; low = 1 g starch/kg BW) by d 4 and 100% by d 11. Fecal samples were obtained at the end of the forage-only period (S0; d -2), and on d 6 (S1) and d 13 (S2) of the treatment period. Cellulolytics, lactobacilli, Group D Gram-positive cocci (GPC), lactate-utilizers and amylolytics were enumerated. Enumeration data were log transformed and analyzed by repeated measures ANOVA. There were sample day × treatment interactions (*P* < 0.0001) for all bacteria enumerated. Enumerations from control horses did not change during the sampling period (*P* > 0.05). All treatments except LO resulted in increased amylolytics and decreased cellulolytics, but the changes were larger in horses fed corn and wheat middlings (*P* < 0.05). Feeding oats resulted in increased lactobacilli and decreased GPC (*P* < 0.05), while corn had the opposite effects. LW had increased lactobacilli and GPC (*P* < 0.05). The predominant amylolytic isolates from HC, LC and LW on S2 were identified by 16S RNA gene sequencing as *Enterococcus faecalis*, but other species were found in oat fed horses. These results demonstrate that starch source can have a differential effect on the equine fecal microbiota.

## Introduction

Cereal grains are often included in equine diets to increase energy. Corn, oat, wheat, and barley starches are similar in that they consist of amylose and amylopectin, but differ in the proportion of those polysaccharides and also in the morphology of the starch granule [1, 2, 3, 4, 5, 6, 7]. Several studies have shown that there are differences among cereal grains in regard to small intestinal digestibility in horses [8, 9, 10, 11, 12].

Starch that bypasses small intestinal digestion can reach the hindgut where it will be fermented by the resident microbiota. Starch fermentation can lead to increased numbers of amylolytic bacteria, including lactobacilli and streptococci, increased lactic acid concentrations, decreased pH, and decreased cellulolytic bacteria [13, 14, 15]. Most notably, *Streptococcus bovis* is considered the major etiological agent of acute acidosis in ruminants and has been found to be numerous in carbohydrate-excess conditions in horses [16, 17, 18, 19, 20]. Furthermore, the bypass of starch to the large intestine and resulting effect on the microbial community has been suggested as one of the mechanisms by which increased concentrate intake elevates the risk for digestive disorders in horses [21].

Both culture-dependent and -independent methods have shown that replacing fiber with starch in equine diets alters the hindgut microbial community [21, 22, 23, 24]. However, studies have not investigated how starch source affects equine hindgut microbiota. We hypothesized that changes to the hindgut bacteria in response to dietary starch would be affected by source of starch. The objective was to compare the effects of adding oats, corn or wheat middlings to a forage-based diet on equine hindgut microbiota.

## Materials and Methods

All procedures were approved by the Institutional Animal Care and Use Committee at the University of Kentucky (protocol 2008–0311). General housing and care of the animals were consistent with the Guide to Care and Use of Agricultural Animals in Research and Teaching [25].

### Animals

Thirty horses (2 to 17 y; 18 mares, 12 geldings; 27 Thoroughbreds, 1 Quarter Horse, 1 Standardbred, 1 Paint ×Thoroughbred cross; 571 +/- 46.9 kg; average body condition score: 6 +/- 0.8) were selected from a resident herd based on a history of no gastrointestinal disease in the preceding 4 mo. Horses were blocked by age and gender into 5 blocks of 6 horses each. Each block of horses was used for 4 wk and all blocks were completed between July and November 2013. During the 4 week study, the horses were housed in individual, partially covered runs with crushed limestone footing (3 × 15 m). Each run contained an automatic water source and the feeding area of the run was equipped with rubber mats, a large plastic tub secured to the wall, and a salt block. Horses were allowed 6 h of turnout per day in dry lot paddocks.

### Experimental design and diets

For the first 2 wk (d -14 to d -1), all horses were fed long stem mid-maturity timothy alfalfa mix hay (Table 1; Creech, Inc., Lexington, KY). Hay was fed at 2% of BW during wk 1 and decreased to 1.67% of BW at the end of wk 2. All horses received this amount of hay for the remainder of the study. The total daily hay allotment was given to each horse at 15:00 in their individual runs immediately following turnout. At the beginning of wk 3, horses in each block were randomly allocated to 1 of 6 treatments: hay only (Control), high corn (HC), high oats (HO), low corn (LC), low oats (LO), and low wheat middlings (LW). Coarsely cracked corn, whole, slightly rolled cleaned oats and pelleted wheat middlings were used (oats and corn: McCauley Bros, Versailles, KY; wheat middlings: Purina-Land 0' Lakes, St. Louis, MO). Prior to each block the oats, cracked corn and pelleted wheat middlings to be used were analyzed for chemical composition using commercial wet chemistry methods (Table 1; Dairy One, Ithaca, NY). The sum of starch and ethanol soluble carbohydrates in each starch source was used to adjust feed amounts to provide either 1 g starch/kg BW (low treatments) or 2 g starch/kg BW (high treatments).

**Table 1:** Chemical composition hay, corn, oats and wheat middlings^1^.

Nutrient^2^	Hay	Cracked Corn	Oats	Wheat Middlings
**DM, %**	91.5	88.2	88.7	91.1
**CP, %**	12.4	10.2	13.1	18.1
**ADF, %**	35.2	5.0	12.2	13.5
**NDF, %**	48.6	9.9	27.0	37.2
**Starch, %**	2.0	67.5	48.2	23.9
**ESC, %**	7.9	2.4	2.6	5.2
**WSC, %**	11.1	2.6	2.6	6.3
**Crude Fat, %**	2.7	4.1	5.7	4.9
**Ca, %**	0.99	0.01	0.09	0.14
**P, %**	0.26	0.32	0.53	1.13

^1^ Dry Matter Basis. Analysis performed by Dairy One, Ithaca NY.

^2^DM–dry matter; CP–crude protein; ADF–acid detergent fiber; NDF–neutral detergent fiber; ESC–ethanol soluble carbohydrates; WSC–water soluble carbohydrates; Ca–calcium; P—phosphorus

Although it would have been desirable to include a high wheat middling treatment, the starch content of the wheat middlings would have necessitated a level of intake above the capacity of the horses. Therefore, a high wheat middling treatment was not included. Consistent with recommended feeding practices [26], the starch sources were introduced to the diet gradually beginning with the afternoon meal (15:00) on d 1 of the treatment period. Horses received 25% of the assigned starch level (high = 2 g starch/kg BW; low = 1 g starch/kg BW) on d 1 to 3, 50% on d 4 to 7, 75% on d 8 to 10 and 100% on d 11 to 14. Horses received an additional ½ of their assigned starch level in the morning prior to turnout (08:00).

Fecal samples were collected at three sampling times. The initial sample was collected at the end of forage-only period, before starch sources were introduced (S0; d -2). The next samples were collected on d 6 (S1) and on d 13 of the starch-feeding period (S2). Feces were collected during defecation as catch samples prior to contact with the floor as to avoid ground contamination.

Immediately following collection on S0, S1, and S2 a subsample (approximately 1 g) of the fresh feces was placed in a pre-weighed sterile Hungate tube for subsequent bacterial enumeration. Once the sample was in the tube, the rubber stopper was replaced, and the tube was purged of air with CO_2_ for 30 sec via tuberculin needle. These fresh samples were then maintained at 37 °C and transported into the laboratory within 2 h of collection. The remaining feces in each collection were used for determination of fecal fluid pH, fecal DM, and short chain fatty acid (SCFA) concentrations. Fecal fluid pH was determined immediately following collection by squeezing a portion of each fecal sample to obtain fecal fluid for pH measurement using a portable pH meter (IQ 150, accuracy ± 0.01 pH units; IQ Scientific Instruments, Loveland, CO). Approximately 100 g of sample was transported in a plastic bag to the laboratory for fecal DM analysis and two 15 mL conical tubes of feces were frozen (-20 °C) for later SCFA analysis.

### Bacterial enumerations

Upon arrival at the laboratory, the Hungate tubes were reweighed and each sample was subjected to 10-fold dilution (w/w) with anaerobic PBS (pH 7.4, N_2_-sparged; 8 g NaCl, 0.2 g KCl, 1.44 g Na_2_PO_4_, 0.24 g KH_2_PO_4_ per L) and mixed by vortex until the suspension was homogenous (~45 sec). The fecal suspensions were then serially diluted (10-fold w/w, anaerobic PBS) in an anaerobic chamber (Coy, Grass Lake, MI, 95% CO_2_, 5% H_2_) for the inoculation of enumeration media.

Total amylolytic bacteria were enumerated in an anaerobic liquid medium with soluble starch from potato [27]. The tubes were incubated (37 °C, 3 d). The highest dilution exhibiting bacterial growth (visual examination) was recorded as the viable number. Lancefield Group D Gram positive cocci (GPC), which include *Enterococcus* spp., *Streptococcus bovis*, and *S*. *equinus* were enumerated on bile esculin azide agar (Enterococcosel; BD, Franklin Lake, NJ). *Lactobacillus* spp. were enumerated on Rogosa SL agar (BD). The plates were incubated aerobically (37 °C, 3 d). Plates with 30 < × < 300 colonies were counted. Black colonies on bile esculin azide agar were counted as GPC. All colonies on Rogosa SL agar were counted as lactobacilli.

Total lactate-utilizing bacteria were enumerated on L-U agar, previously developed by Mackie and Heath (1979) [28]. The plates were incubated in an anaerobic chamber (37 °C, 5 d). Plates with 30 > × < 300 colonies were counted. All colonies on L-U agar were counted as lactate-utilizing bacteria.

Cellulolytic bacteria were enumerated in an anaerobic defined liquid medium with Whatman #1 filter paper strips as the growth substrate [29]. Incubations were carried out under anaerobic conditions at 37 °C for 10 d. Growth was evaluated daily by dissolution of the cellulose substrate and microscopy. The final dilution exhibiting dissolution of cellulose on d 10 was recorded as the viable number of cellulolytic bacteria.

### Isolation and characterization of predominant amylolytic bacteria

Solid amylolytic medium was made for bacterial isolation by preparing with agar (15 mg/mL) in Petri plates. The plates were poured, inoculated, and incubated (37°C, 24 h) in an anaerobic chamber (Coy Labs, Grass Lake, MI, USA) with a 95% CO_2_ and 5% H_2_ atmosphere.

Samples (200 μL) from the highest dilutions of total amylolytic bacteria were used to streak anaerobic solid amylolytic selective medium for isolation of amylolytic bacteria. Colonies were then picked and routinely passaged in liquid amylolytic medium (37°C, 24 to 48 h). Colonies were selected based on differences in morphology and microscopy. Pure cultures were cryopreserved for later identification and characterization (-80°C).

Isolates were characterized by light microscopy and Gram stain. They were further characterized for substrate utilization in the basal medium with glucose, inulin, or esculin as the substrate. Blood hemolysis was determined by amending solid basal medium with glucose (4 mg mL^-1^) and defibrinated horse blood (5%; Quad Five, Ryegate, MT, USA). The plates were poured, inoculated, and incubated (37°C, 24 h) in an anaerobic chamber as described above. The isolate that appeared to be *E*. *coli* was repeatedly re-isolated, and grown aerobically on *E*. *coli* selective & differential agar (CHROMagar™ E. coli; Chromagar, Paris). It was grown in the presence of monensin (10 μM) to ensure that there were no Gram-positive contaminants.

The phylogenetic identities were determined by 16S RNA gene sequencing. Briefly, DNA was extracted from each isolate (lysozyme and achromopeptidase with ethanol precipitation), amplified using PCR universal 16S primers (Integrated DNA Technologies, Coralville, IA, USA; 5’-AGAGTTTGATCCTGGCTCAG, 3’–ACGGCTACCTTGTTACGACTT) and then sequenced by the University of Kentucky, Advanced Genetic Technologies Center (Lexington, KY) using an ABI 3730 DNA Analyzer (Applied Biosystems, Norwalk, CT). Sequences were aligned and analyzed using Geneious (v. 5.1; [30]). The closest phylogenetic relatives of the isolates were determined using a BLAST search of GenBank [31].

### SCFA analyses

Fecal samples were thawed, mixed with water (50% w/w) and clarified in a microcentrifuge (21,000 × *g*, 2 min). Particulate matter was removed from the supernatants with microcentrifuge prep columns (Amicon Ultra-4 Centrifugal Filter; Millipore). Short chain fatty acids were quantified using an HPLC method previously used by our research group and others (27, 32). The instrument was a Dionex HPLC (Sunnyvale, CA) equipped with an anion exchange column (Aminex HP-87H; Bio-Rad, Hercules, CA), refractive index (Shodex/Showa Denko, Kanagawa, Japan) and UV detector (Dionex, Sunnyvale, CA). The column was operated at 50°C with a 0.4 ml/min flow rate, and a H_2_SO_4_ (0.17N) mobile phase. The water dilution step was accounted for in the results.

### Statistical Analyses

Enumeration data were normalized by log_10_ transformations prior to statistical analyses. Values in figures and tables are presented as true means unless otherwise noted. One horse assigned to HO was injured in a paddock accident and was removed from the study. Consequently, the effect of low starch intake (*n* = 5 / treatment) and the effect of high starch intake (*n* = 4 / treatment) were analyzed separately. Data were analyzed using the MIXED procedure of SAS as a randomized block design with a repeated measures treatment design (v. 9.3, SAS Institute, Inc. Cary, NY). The class statement included horse, treatment (starch source), sample day and block. The model statement included the response variable of interest as the dependent variable and treatment, sample day, block and the interaction between treatment and sample day as fixed effects. The Kenward-Roger method was used to compute the denominator degrees of freedom for the fixed effects and the repeated statement requested the autoregressive (ar-1) covariance structure. Means were separated using the lsmeans statement with the pdiff option when the F-test for the main effect of interest (treatment, sample day or treatment × sample day interaction) was significant (*P* < 0.05). Simple linear regression was used to identify relationships among the number of lactobacilli and the number of total amylolytic bacteria as well as between the number of lactobacilli and the number of GPC.

For statistical comparison of predominant amylolytic bacteria isolated from each treatment, isolate species were individually assigned a 0 (not predominant isolate) or 1 (predominant isolate) for each observation. The strength of association between treatment and isolate species within sample day and between sample days and isolate species within treatment were evaluated by Chi-square analysis using the FREQ procedure of SAS (v. 9.3, SAS Institute, Inc. Cary, NY, USA). The cellchi2 option was used to display the contribution of each individual comparison to the total Pearson chi-square statistic. Statistical significance was set at *P* < 0.05 and a trend at 0.05 < *P* < 1.0.

## Results

### Fecal pH, fecal DM and SCFA analyses

For S0 (when all horses were fed only forage), mean fecal fluid pH was 7.79 (range, 7.28 to 8.07; σ, ± 0.20) and mean fecal DM was 22.91% (range, 19.15% to 24.67%, σ, ± 1.38%). Feeding starch at the high level of intake resulted in treatment × sample day interactions for fecal fluid pH (*P* = 0.0052) and fecal DM (Table 2; *P* < 0.0001). Both variables remained consistent across sample day in control horses (*P* > 0.05). Fecal fluid pH decreased in horses fed HC (*P* < 0.05) but was unchanged in horses fed HO (*P* > 0.05). Fecal DM decreased in horses fed HC, but increased in horses fed HO (*P* < 0.05). There were treatment × sample day interactions for both fecal fluid pH (*P* < 0.0001) and fecal DM (*P* = 0.003) in horses fed low starch intakes as well (Table 3). For both variables, values were consistent across sample day for control and LO (*P* > 0.05) but decreased in LC and LW (*P* < 0.05). There were no sample day, treatment or treatment × sample day effects for fecal acetate (high: 107.6, 1, ± 25.2; low: 111.9, 5, ± 12.3), propionate (high: 26.7, 2, ± 10.3; low: 27.5, 2, ± 6.3) or butyrate (high: 23.7, (, ± 37.4; low: 12.8, 7, ± 4.4) in horses fed either the high or low intakes of starch (*P* > 0.05; data not shown). No lactate was detected in any fecal sample over the course of the study.

**Table 2:** Effect of high oats and high corn diets on fecal pH and DM.

		SO^1^	S1	S2	Pooled SEM	*P*-value^2^
**Fecal pH**					**0.12**	**0.0052**
	Control^3^	7.8^a^^,^^α^	7.9^a^^,^^α^	8.0^a^^,^^α^		
	HO	7.8^a^^,^^α^	7.7^a^^,^^α^	8.0^a^^,^^α^		
	HC	7.8^a^^,^^α^	6.9^b^^,^^β^	7.1^b^^,^^β^		
**Fecal DM, %**					**0.37**	**0.0001**
	Control	22.4^a^^,^^α^	23.0^a^^,^^α^	23.2^a^^,^^α^		
	HO	23.0^a^^,^^α^	26.1^b^^,^^β^	26.0^b^^,^^β^		
	HC	21.7^a^^,^^α^	19.9^b^^,^^γ^	18.6^c^^,^^γ^		

^1^Sample day–S0, end of the forage-only period before horses had received any starch. S1, 6 d after starch was introduced to the diet (starch intake at 50% of the final intended rate). S3, 13 d after starch was introduced to the diet (starch intake at 100% of the final intended rate).

^2^Sample day × treatment (starch source) interaction

^3^Treatment (starch source)–Control, hay only (*n* = 4), HO, high oats (2 g starch/kg BW; *n* = 4), HC, high corn (2 g starch/kg BW; *n* = 4).

^a,b,c^Means lacking a common English letter are different between sample days (rows; *P* < 0.05).

^α, β, γ^ Means lacking a common Greek letter are different between starch sources within samples days (columns; *P* < 0.05).

**Table 3:** Effect of low oats, low corn and low wheat middling diets on fecal pH and DM.

		SO^1^	S1	S2	Pooled SEM	*P*-value^2^
**Fecal pH**					**0.08**	**0.0001**
	Control^3^	7.8^a^^,^^α^	7.9^a^^,^^α^	8.0^a^^,^^α^		
	LO	7.8^a^^b^^,α^	7.5^b^^,^^β^	7.8^a^^,^^α^		
	LC	7.8^a^^,^^α^	6.9^b^^,^^γ^	7.1^b^^,^^β^		
	LW	7.8^a^^,^^α^	6.5^b^^,^^δ^	6.5^b^^,^^γ^		
**Fecal DM, %**					**0.5**	**0.003**
	Control	22.8^a^^,^^α^	23.3^a^^,^^α^	23.2^a^^,^^α^		
	LO	22.4^a^^,^^α^	23.1^a^^,^^α^	23.3^a^^,^^α^		
	LC	23.6^a^^,^^α^	22.3^a^^,^^α^	21.6^b^^,^^β^		
	LW	23.4^a^^,^^α^	23.1^a^^,^^α^	20.6^b^^,^^γ^		

^1^Sample day–S0, end of the forage-only period before horses had received any starch. S1, 6 d after starch was introduced to the diet (starch intake at 50% of the final intended rate). S3, 13 d after starch was introduced to the diet (starch intake at 100% of the final intended rate).

^2^Sample day × treatment (starch source) interaction

^3^Treatment (starch source)–Control, hay only (*n* = 4); LO, low oats (1 g starch/kg BW; *n* = 4), LC, low corn (1 g starch/kg BW; *n* = 4); LW, low wheat middlings (1 g starch/kg BW, *n* = 4)

^a, b^ Means lacking a common English letter are different between sample days (rows; *P* < 0.05).

^α, β, γ, δ^ Means lacking a common Greek letter are different between starch sources within samples days (columns; *P* < 0.05).

### Enumeration of amylolytic bacteria

**Fig 1:**
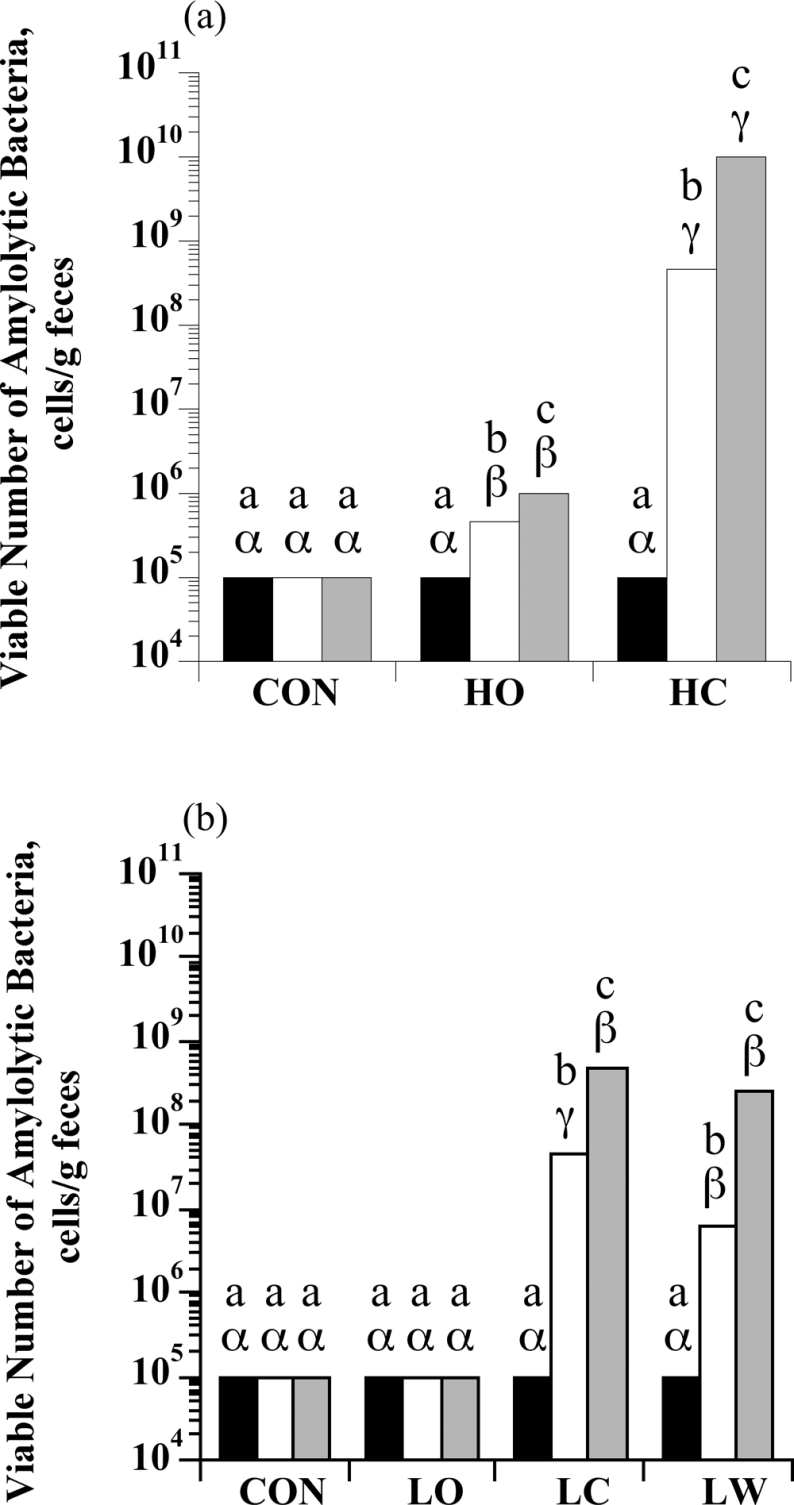
The effect of dietary starch source (corn, oats, and wheat middlings) on the viable number of total amylolytic bacteria in equine feces. (a) Horses were assigned to treatments: hay only (CON; *n* = 4), high oats (HO; *n* = 4; 2 g starch/kg BW), or high corn (HC; *n* = 4; 2 g starch/kg BW). (b) Horses were assigned to treatments: hay only (CON; *n* = 5), low oats (LO; *n* = 5; 1 g starch/kg BW), low corn (LC; *n =* 5; 1 g starch/kg BW), or low wheat middlings (LW; *n* = 5; 1 g starch/kg BW). An initial sample was obtained at the end of the forage-only feeding period (S0; black bars) before starch sources were introduced. Additional samples were taken on d 6 when horses were receiving 50% of the assigned starch source (S1; open bars) and on d 13 when they were receiving 100% of the assigned starch source (S2; grey bars). The enumerations were performed in anaerobic liquid media with soluble starch as the growth substrate. The tubes were incubated (37°C, 3 d), and the final dilution exhibiting bacterial growth was recorded as the viable number. Means lacking a common English letter are different within sample day (*P* < 0.05). Means lacking a common Greek letter are different between sample days within a treatment (*P* < 0.05); (a) Treatment: *P* < 0.0001, sample day: *P* < 0.0001, and treatment × sample day: *P* < 0.0001; Pooled SEM: treatment = 0.0731, sample day = 0.0780, and treatment × sample day = 0.1351 (log_10_ transformed); (b) Treatment: *P* < 0.0001, sample day: *P* < 0.0001, and treatment × sample day: *P* < 0.0001; Pooled SEM: treatment = 0.1109, sample day = 0.0794, and treatment × sample day = 0.1588 (log_10_ transformed).

There was a treatment × sample day interaction for amylolytic bacteria enumerations with high starch intake (*P* < 0.0001; Fig 1A). Amylolytic bacteria increased in both HC and HO horses, but the magnitude of this increase was starch source dependent. For S2, horses that received HO had 10-fold more amylolytic bacteria than horses fed hay only (*P* < 0.05). In contrast, horses fed HC had 100,000-fold more amylolytic bacteria than control horses for S2 (*P* < 0.05). There was also a treatment × sample day interaction for amylolytic bacteria enumerations with low starch intake (*P* < 0.0001; Fig 1B). Both LC and LW treatments increased total amylolytic bacteria in comparison to control (10,000-fold; *P* < 0.05), whereas the number of amylolytic bacteria was unchanged in horses fed LO (*P* > 0.05).

### Isolation, identification and characterization of predominant amylolytic bacteria

The identity of each isolate is presented by experimental block and sample time in Table 4. Further characterization of the isolates is presented in a supplementary Table (S1 Table). Some amylolytic isolates did not survive cryopreservation or had poor laboratory viability, thus, an isolate was not obtained from every sample. No amylolytic isolates were obtained from block 2. However, at least 3 amylolytic isolates (61 total isolates) were characterized and identified for each treatment and sampling time point. The majority of amylolytic bacteria isolated were Gram-positive cocci that occurred in chains (58/61 isolates; Table 4, S1 Table). All 61 isolates were able to utilize both glucose and inulin as a primary energy source and were non-hemolytic on horse blood agar (excluding 1 *S*. *bovis* isolate from LO d13 block 3, which was alpha-hemolytic). However, sequence (16S) analysis revealed that the identity of the isolates varied greatly depending on treatment. Overall, most (68%) of the amylolytic isolates obtained over the course of the study grouped phylogenetically with genus *Enterococcus* with 93% of those belonging to the species *E*. *faecalis*. Furthermore, 52% of amylolytic isolates obtained at the end of the forage-only period (S0; hay only diet) were identified as *E*. *faecalis*.

**Table 4:** Closest phylogenetic relative of the equine amylolytic bacteria isolates (> 97% 16S gene sequence identity).

	Blk	S0	S1	S2
**CON**	**1**	*Enterococcus faecalis*	-	*Enterococcus faecalis*
	**3**	*Enterococcus faecalis*	*Enterococcus faecalis*	*Enterococcus faecalis*
	**4**	*Enterococcus faecalis*	*Actinobacillus succinogenes*	-
	**5**	*Enterococcus faecalis*	*Enterococcus faecalis*	*Enterococcus faecalis*
**HC**	**1**	*Enterococcus faecalis*	*Enterococcus avium*	*Enterococcus faecalis*
	**3**	*Streptococcus bovis*	-	*Enterococcus faecalis*
	**4**	*Streptococcus macedonicus*	*Enterococcus faecalis*	-
	**5**	*Escherichia coli*	*Enterococcus faecalis*	*Enterococcus faecalis*
**HO**	**1**	*Enterococcus faecalis*	*Streptococcus bovis*	*Lactococcus lactis*
	**3**	*Enterococcus faecalis*	*Enterococcus faecalis*	*Enterococcus aviumStreptococcus bovis*
	**4**	-	*Enterococcus faecalis*	-
	**5**	*Streptococcus macedonicus*	-	*Clostridium sordellii*
**LC**	**1**	*Lactococcus lactis*	*Enterococcus avium*	*Enterococcus faecalis*
	**3**	*Enterococcus faecalis*	*Streptococcus bovis*	*Enterococcus faecalis*
	**4**	-	*Enterococcus faecalis*	*Enterococcus faecalis*
	**5**	*Streptococcus macedonicus*	*Enterococcus faecalis*	*-*
**LO**	**1**	*Streptococcus bovis*	*Lactococcus lactis*	*Enterococcus avium*
	**3**	*Enterococcus faecalis*	*Streptococcus criceti*	*Streptococcus bovis*
	**4**	-	*Enterococcus faecalis*	*Enterococcus faecalis*
	**5**	*Enterococcus faecalis*	*Enterococcus faecalis*	*-*
**LW**	**1**	*Actinobacillus succinogenes*	*Enterococcus faecalis*	*Enterococcus faecalis*
	**3**	*Streptococcus bovis*	*Enterococcus faecalis*	*Enterococcus faecalis*
	**4**	*Streptococcus macedonicus*	*Enterococcus faecalis*	*Enterococcus faecalis*
	**5**	*Enterococcus faecalis*	-	*Enterococcus faecalis*

- indicates isolate was not identified and characterized

CON (hay only), HC (high corn), HO (high oats), LC (low corn), LO (low oats), LW (low wheat); high = 2 g starch/kg BW, low = 1 g starch/kg BW

S0 (hay only), S1 (d 6; 50% final starch intake), S2 (d 13; 100% final starch intake)

There was an association between treatment and the predominance of *E*. *faecalis* on S1 (50% starch; *P* = 0.0617) and S2 (100% starch; *P* = 0.0082). *Enterococcus faecalis* was the most common predominant bacterium in both corn and wheat middling fed horses (S1, 7/10 *E*. *faecalis*; S2, 10/10 *E*. *faecalis*). In contrast, oat fed horses had a variety of predominant amylolytic bacteria on S1 and S2 (7/14 *Enterococcus* spp., 4/14 *Streptococcus* spp., 2/14 *Lactococcus lactis*, 1/14 *Clostridium sordellii*).

Furthermore, there was an association between sample day and the predominance of *E*. *faecalis* within the HC (*P* = 0.0764), HO (*P* = 0.0941), LC (*P* = 0.0022) and LW (*P* = 0.0271) treatments. In corn fed horses (both HC and LC), the frequency with which *E*. *faecalis* was isolated increased with increasing starch intake (HC: S0, 2/7 *E*. *faecalis*; S1, 57% *E*. *faecalis*; S2, 100% *E*. *faecalis*), and a similar trend was also observed in wheat middling fed horses (S0, 25% *E*. *faecalis*; S1 and S2, 100% *E*. *faecalis*). In contrast, the frequency of *E*. *faecalis* isolation decreased with increasing starch intake in HO horses. On S2, *E*. *faecalis* was not isolated from any horse fed the HO diet.

### Enumeration of GPC bacteria

**Fig 2:**
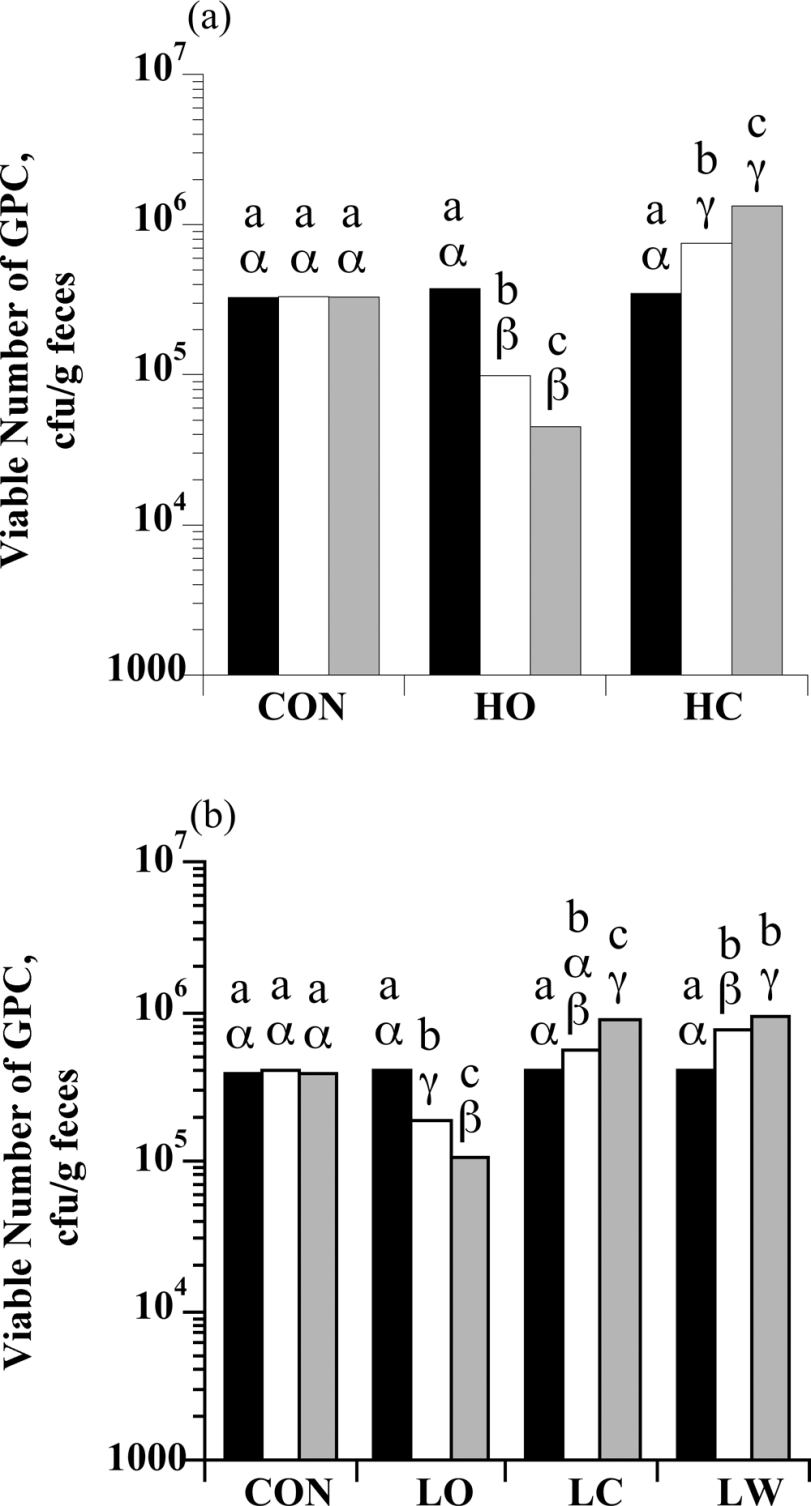
The effect of dietary starch source (corn, oats, and wheat middlings) on the viable number of Group D Gram-positive cocci (GPC; enterococci and *Streptococcus bovis/equinus*) in equine feces. (a) Horses were assigned to treatments: hay only (CON; *n* = 4), high oats (HO; *n* = 4; 2 g starch/kg BW), or high corn (HC; *n* = 4; 2 g starch/kg BW). (b) Horses were assigned to treatments: hay only (CON; *n* = 5), low oats (LO; *n* = 5; 1 g starch/kg BW), low corn (LC; *n* = 5; 1 g starch/kg BW), or low wheat middlings (LW; *n* = 5; 1 g starch/kg BW). An initial sample was obtained at the end of the forage-only feeding period (S0; black bars) before starch sources were introduced. Additional samples were taken on d 6 when horses were receiving 50% of the assigned starch source (S1; open bars) and on d 13 when they were receiving 100% of the assigned starch source (S2; grey bars). Means lacking a common English letter are different within sample day point (*P* < 0.05). Means lacking a common Greek letter are different between sample days within a treatment (*P* < 0.05); (a) Treatment: *P* = 0.0026, sample day: *P* = 0.9259, and treatment × sample day: *P* < 0.0001; Pooled SEM: treatment = 0.0948, sample day = 0.0713, and treatment × sample day = 0.1234 (log_10_ transformed); (b) Treatment: *P* = 0.0002, sample day: *P* = 0.0879, and treatment × sample day: *P*< 0.0001; Pooled SEM: treatment = 0.0642, sample day = 0.0423, and treatment × sample day = 0.0859 (log_10_ transformed).

At the end of the forage-only period, when all horses were fed a hay only diet (S0), the number of GPC bacteria observed in the feces of individual horses ranged from 2.05 × 10^4^ to 6.55 × 10^5^ cfu/g feces. There was a treatment × sample day interaction for fecal GPC enumerations with high starch intake (*P* < 0.0001; Fig 2A). The viable number of GPC in HC horses increased (*P* < 0.05); however, the viable number of GPC in HO horses decreased (*P* < 0.05). There was also a treatment × sample day interaction for GPC enumerations with low starch intake (*P* < 0.0001; Fig 2B). Viable numbers of GPC increased in both LW and LC (*P* < 0.05), but decreased in horses fed LO (*P* < 0.05).

### Enumeration of lactobacilli

**Fig 3:**
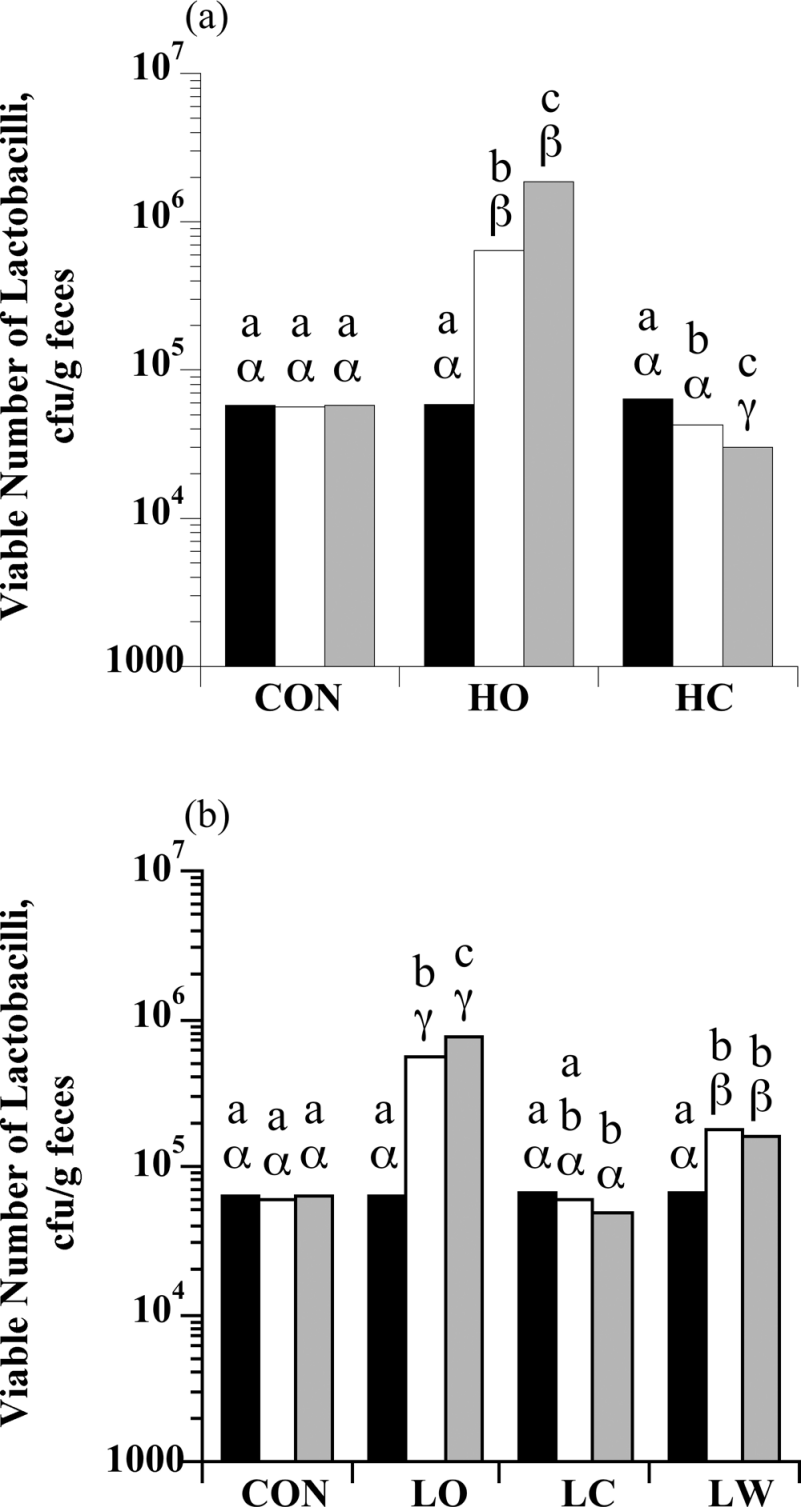
The effect of dietary starch source (corn, oats, and wheat middlings) on the viable number of lactobacilli in equine feces. (a) Horses were assigned to treatments: hay only (CON; *n* = 4), high oats (HO; *n* = 4; 2 g starch kg BW^-1^), or high corn (HC; *n* = 4; 2 g starch/kg BW). (b) Horses were assigned to treatments: hay only (CON; *n* = 5), low oats (LO; *n* = 5; 1 g starch/kg BW), low corn (LC; *n* = 5; 1 g starch/kg BW), or low wheat middlings (LW; *n* = 5; 1 g starch/kg BW). An initial sample was obtained at the end of the forage-only feeding period (S0; black bars) before starch sources were introduced. Additional samples were taken on d 6 when horses were receiving 50% of the assigned starch source (S1; open bars) and on d 13 when they were receiving 100% of the assigned starch source (S2; grey bars). Means lacking a common English letter are different within sample day (*P* < 0.05). Means lacking a common Greek letter are different between sample days within a treatment (*P* < 0.05); (a) Treatment: *P* < 0.0001, sample day: *P* < 0.0001, and treatment × sample day: *P* < 0.0001; Pooled SEM: treatment = 0.0481, sample day = 0.0315, and treatment × sample day = 0.0546 (log_10_ transformed); (b) Treatment: *P* < 0.0001, sample day: *P* < 0.0001, and treatment × sample day: *P* < 0.0001; Pooled SEM: treatment = 0.0360, sample day = 0.0237, and treatment × sample day = 0.0473 (log_10_ transformed).

The number of lactobacilli in feces from horses ranged from 2.40 × 10^4^ to 9.65 × 10^4^ cfu/g feces in S0. There was a treatment × sample day interaction for lactobacilli enumerations with high starch intake (*P* < 0.0001; Fig 3A). The number of fecal lactobacilli did not change during the sampling period in control horses (*P* > 0.05), but decreased in HC horses (*P* < 0.05). In contrast, the number of lactobacilli increased (*P* < 0.05) in HO horses. There was also a treatment × sample day interaction for fecal lactobacilli enumerations with low starch intake (*P* = 0.0013; Fig 3B).

In this case, the LC diet resulted in decreased numbers of viable lactobacilli, compared to controls while both LO and LW diets promoted the growth of lactobacilli (*P* < 0.05). Furthermore, within fecal samples from S2, there were negative correlations between the viable number of lactobacilli and total amylolytic bacteria (r = -0.97) and between lactobacilli and GPC (r = -0.99; data not shown).

### Enumeration of lactate-utilizing bacteria

**Fig 4:**
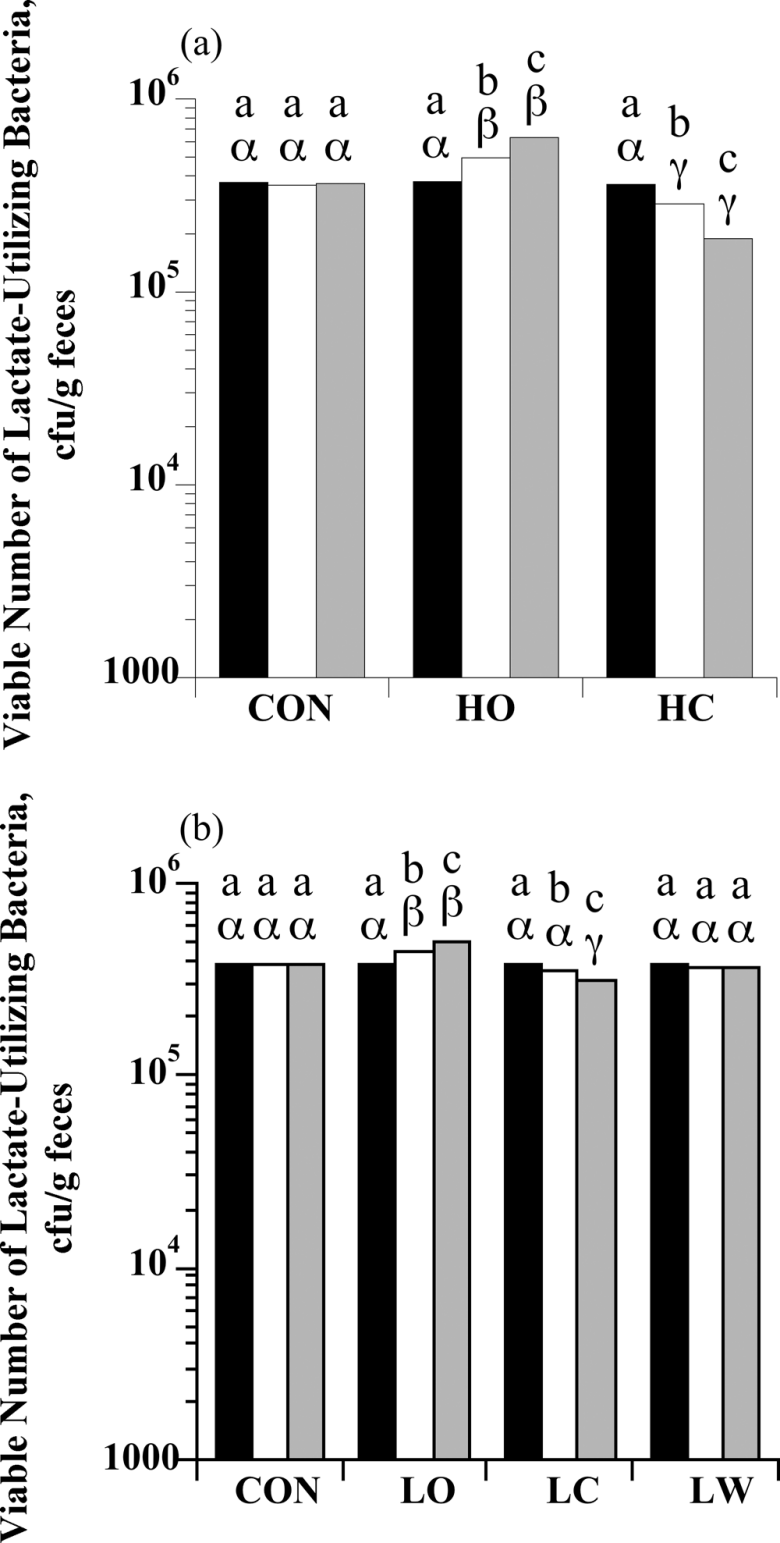
The effect of dietary starch source (corn, oats, and wheat middlings) on the viable number of total lactate-utilizing bacteria in equine feces. (a) Horses were assigned to treatments: hay only (CON; *n* = 4), high oats (HO; *n* = 4; 2 g starch/kg BW), or high corn (HC; *n* = 4; 2 g starch/kg BW). (b) Horses were assigned to treatments: hay only (CON; *n* = 5), low oats (LO; *n* = 5; 1 g starch/kg BW), low corn (LC; *n* = 5; 1 g starch/kg BW), or low wheat middlings (LW; *n* = 5; 1 g starch/kg BW). An initial sample was obtained at the end of the forage-only feeding period (S0; black bars) before starch sources were introduced. Additional samples were taken on d 6 when horses were receiving 50% of the assigned starch source (S1; open bars) and on d 13 when they were receiving 100% of the assigned starch source (S2; grey bars). Means lacking a common English letter are different within sample day point (*P* < 0.05). Means lacking a common Greek letter are different between sample days within a treatment (*P* < 0.05); (a) Treatment: *P* = 0.0001, sample day: *P* = 0.3902, and treatment × sample day: *P* < 0.0001; Pooled SEM: treatment = 0.02121, sample day = 0.0165, and treatment × sample day = 0.0287 (log_10_ transformed); (b) Treatment: *P* = 0.0002, sample day: *P* = 0.9747, and treatment × sample day: *P* < 0.0001; Pooled SEM: treatment = 0.0114, sample day = 0.0073, and treatment × sample day = 0.0145 (log_10_ transformed).

The number of total lactate-utilizing bacteria in S0 feces of individual horses ranged from 3.00 × 10^5^ to 4.55 × 10^5^ cfu/g. There was a treatment × sample day interaction for total lactate-utilizing bacteria enumerations with high starch intake (*P* < 0.0001; Fig 4A). The number of lactate-utilizing bacteria increased in horses fed HO (*P* < 0.05), but decreased in horses fed HC (*P* < 0.05), while no change in numbers occurred in horses fed the control diet (*P* > 0.05). There was also a treatment × sample day interaction for total lactate-utilizing bacteria with low starch intake (*P* < 0.0001; Fig 4B). Feeding the LO diet promoted the growth of lactate-utilizing bacteria with the opposite effect observed in LC horses (*P* < 0.05, in both cases). No changes in lactate-utilizing bacteria were observed in control or LW horses (*P* > 0.05).

### Enumeration of cellulolytic bacteria

**Fig 5:**
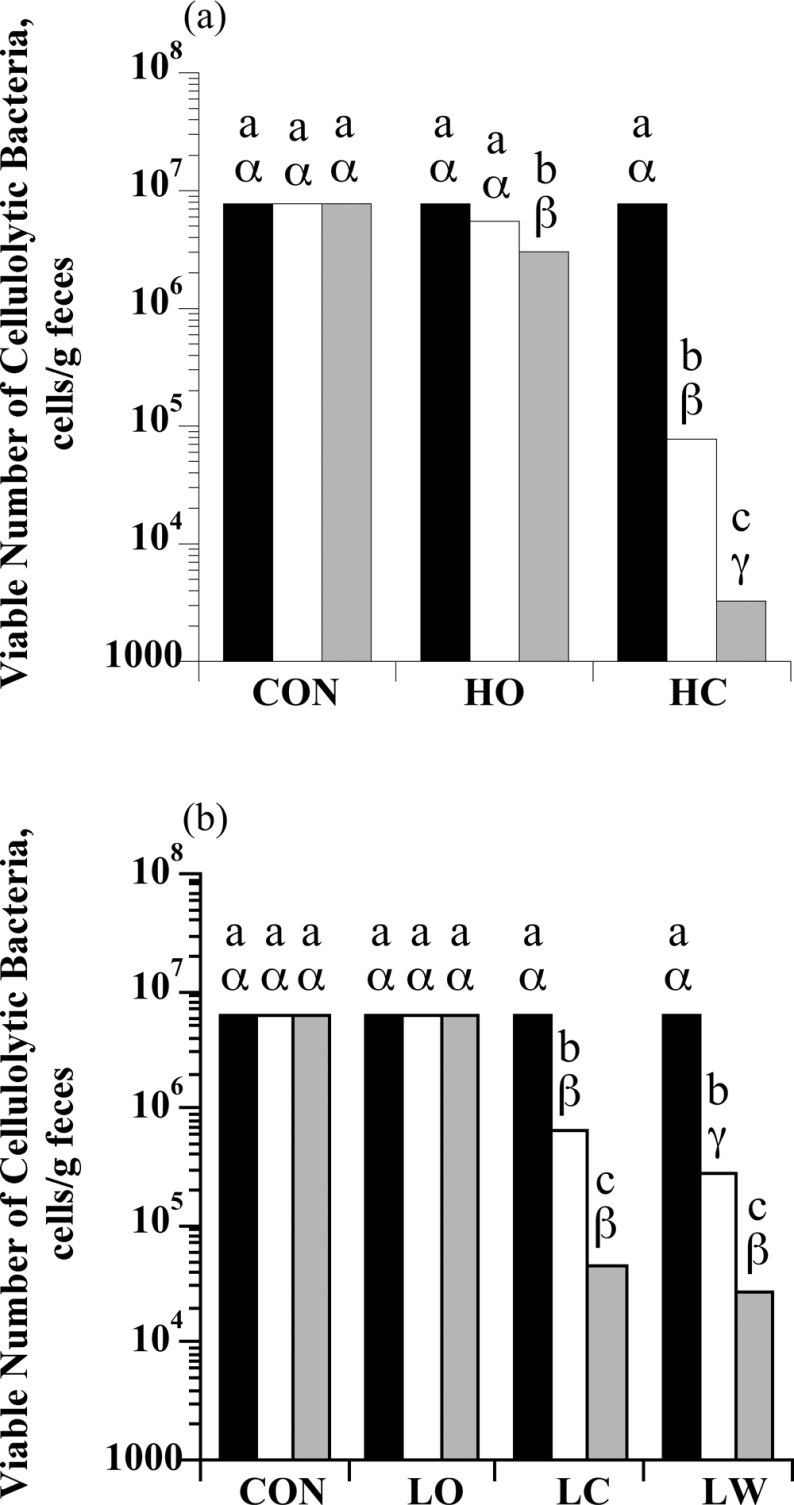
The effect of dietary starch source (corn, oats, and wheat middlings) on the viable number of total cellulolytic bacteria in equine feces. (a) Horses were assigned to treatments: hay only (CON; *n* = 4), high oats (HO; *n* = 4; 2 g starch/kg BW), or high corn (HC; *n* = 4; 2 g starch/kg BW). (b) Horses were assigned to treatments: hay only (CON; *n* = 5), low oats (LO; *n* = 5; 1 g starch/kg BW), low corn (LC; *n* = 5; 1 g starch/kg BW), or low wheat middlings (LW; *n* = 5; 1 g starch/kg BW). An initial sample was obtained at the end of the forage-only feeding period (S0; black bars) before starch sources were introduced. Additional samples were taken on d 6 when horses were receiving 50% of the assigned starch source (S1; open bars) and on d 13 when they were receiving 100% of the assigned starch source (S2; grey bars). Means lacking a common English letter are different within sample day (*P* < 0.05). Means lacking a common Greek letter are different between sample days within a treatment (*P* < 0.05); (a) Treatment: *P* < 0.0001, sample day: *P* < 0.0001, and treatment × sample day: *P* < 0.0001; Pooled SEM: treatment = 0.1059, sample day = 0.0935, and treatment × sample day = 0.1620 (log_10_ transformed); (b) Treatment: *P* < 0.0001, sample day: *P* < 0.0001, and treatment × sample day: *P* < 0.0001; Pooled SEM: treatment = 0.0894, sample day = 0.0614, and treatment × sample day = 0.1227 (log_10_ transformed).

At the end of the forage-only period (S0), the cellulolytic bacteria in the feces of individual horses ranged from 10^6^ to 10^7^ cfu/g. The number of cellulolytic bacteria remained constant in horses fed the control diet, but decreased in horses fed HC and HO (treatment × sample day: *P* < 0.0001; Fig 5A). Furthermore, while the number of cellulolytic bacteria decreased from S0 to S2 in both HC and HO horses, the magnitude of the decrease was starch source dependent. Horses fed HO had < 10-fold fewer viable cellulolytic bacteria than control horses in S2 (*P* < 0.05). In contrast, HC horses had 1,000-fold fewer cellulolytic bacteria than control in S2 feces (*P* < 0.05). There was also a treatment × sample day interaction for total cellulolytic bacteria enumerations with low starch intake (*P* < 0.0001; Fig 5B). The number of cellulolytic bacteria in the feces of horses fed LC and LW decreased (*P* < 0.05), but remained constant in horses fed LO or the control diet (*P* > 0.05).

## Discussion

Starch source has been shown to affect the extent of starch bypass to the equine hindgut [8, 9, 10, 11, 12]. We hypothesized that starch source would also affect changes to the gastrointestinal microbial community that are induced when grain is added to a forage-based diet.

At the lower level of starch intake, the number of amylolytic bacteria was increased by corn and wheat middlings, but not by oats. At the higher level of starch intake, both oats and corn increased the number of amylolytic bacteria, but the magnitude of the increase was much greater for horses fed corn. The differential responses to corn and oats are consistent with previous studies that have demonstrated that corn starch is less susceptible to enzymatic digestion in the equine small intestine and, therefore more likely to be available for fermentation in the large intestine, than oat starch [8, 12]. Conversely, in a previous *in vitro* study utilizing an equine fecal cell suspension model that did not account for foregut enzymatic digestion, a similar response was observed with corn and oat fermentation [32]. Therefore, differences observed between starch sources on the fecal microbiota in the current study could be partially explained by direct effects of starch source on hindgut microorganisms.

In addition to affecting the number of total amylolytic bacteria, starch source affected the number of lactobacilli and GPC bacteria. At both the high and low level of starch intake, corn resulted in an increase in GPC bacteria, while adding oats to the diet resulted in a decrease in GPC bacteria. As observed for total amylolytic bacteria, the response of the GPC bacteria to wheat middlings was more similar to the response observed with corn than with oats. The proliferation of GPC has previously been reported in response to increasing the amount of barley starch in the diet [13, 14, 15]. Barley is generally considered to be similar to corn in regard to small intestinal starch digestibility [33]. The GPC numbers in these latter studies were reported as streptococci. However, bile esculin azide agar also permits the growth of other GPC. Thus, we report the results as GPC, but the results are comparable to “streptococci” in the previous studies. Streptococci have also been shown to proliferate in response to increasing dietary oligofructose [19], but an increase in streptococci in response to feeding oats has been less consistently reported. Kern *et al*. (1973) did not observe an increase in cecal streptococci when oats were added to hay-based diets [22]. However, Willing *et al*. (2009) reported that total lactic acid producing bacteria, including streptococci, increased in the feces when oats were substituted for forage in the diet of horses [15].

The number of lactobacilli in the feces of horses fed HO, LO and LW increased during the sampling period. Conversely, the number of lactobacilli in the feces of horses fed corn decreased during the sampling period. Consequently, it appears that although all starch sources stimulated an increase in the number of total amylolytic bacteria, there were differences in which amylolytic bacteria proliferated in response to each starch source. These results are consistent with a previous *in vitro* study that demonstrated that oats promoted lactobacilli proliferation while corn promoted GPC proliferation in equine fecal cell suspensions [32].

In the current study, when the viable number of GPC and lactobacilli were combined the total viable number was approximately equal to the viable number of total amylolytic bacteria observed in hay and oat fed horses. However, lactobacilli and GPC did not account for 99.99% of the amylolytic bacteria in horses fed corn and wheat middlings, which increased by as much as 100,000-fold when grain feeding began. These observations resulted in the hypothesis that particular predominant amylolytic bacteria would be selected for by starch source. To investigate this hypothesis, the predominant fecal amylolytic bacteria isolated from the horses in this study were identified and characterized.

Although most isolates were similar in morphology, Gram-stain, and substrate utilization, sequence (16S) analysis revealed that the identity of the isolates varied greatly depending on treatment. Most (64%) of the amylolytic isolates were identified as *E*. *faecalis*, including 52% of the amylolytic isolates in SO (hay-only diet) fecal samples. These results are the first, to our knowledge, that identify *E*. *faecalis* as a predominant amylolytic bacterium in equine feces. Furthermore, there were associations between treatment, sample day and the predominance of *E*. *faecalis*. *E*. *faecalis* was the predominant amylolytic bacterium in both corn and wheat middling fed horses with a greater frequency of isolation observed with increasing starch intake. In contrast, oat fed horses had a variety of predominant amylolytic bacteria with the frequency of *E*. *faecalis* isolation decreasing with increasing starch intake. These results are also consistent with a previous *in vitro* study that identified *E*. *faecalis* as the predominant amylolytic in corn and wheat fermentations but not in oat fermentations [32].

All *E*. *faecalis* isolates in the current study were able to utilize esculin as a sole carbon source, which is typical of the species [34]. However, *E*. *faecalis* isolated from HC, LC and LW horses on S1 and S2 were not enumerable on bile esculin azide agar, indicating that these isolates are susceptible to the antimicrobial action of bile and azide. Bile esculin azide agar is routinely used in equine studies for the selective enumeration of amylolytic bacteria [8, 18, 24, 32]. However, based on the results of the current study, bile esculin azide agar is not a sufficient medium for detecting predominant amylolytic bacteria under certain conditions. A non-selective media type with starch as the sole growth substrate, like the basal medium + soluble starch utilized in the current study, might enhance detection of amylolytic bacteria from equine feces.

*Enterococcus faecalis*, previously classified as *Streptococcus faecalis*, is a Gram-positive, non-spore forming, facultative anaerobic bacterium that is indigenous to the gastrointestinal tract [34, 35, 36]. Enterococci are among the most common nosocomial pathogens [37]. They produce several virulence factors and possess a broad spectrum of intrinsic and transferable antibiotic resistance mechanisms [37]. *E*. *faecalis* is extremely pH-tolerant, oxygen-tolerant, has the ability to produce bacteriocins and other small antimicrobial molecules, and has a broad metabolic capacity (saccharolytic, proteolytic and lipolytic functionality) allowing it to grow in most environments [36, 38]. Additionally, *E*. *faecalis* has the ability to produce amines and proteinases and could, therefore, play an important role in the development of several starch-overload related health conditions in horses [39].

Previous research has implicated *S*. *bovis*/*equinus* as the major etiological agent of acute rumen acidosis (lactic acidosis) and several carbohydrate overload-related health conditions in horses because of its prevalence in carbohydrate excess conditions and its ability to produce amines and proteinases [16, 18, 19, 20, 40]. However, only 11% of amylolytic isolates in the current study phylogenetically grouped with *S*. *bovis*. The horses in the current study were gradually adapted over a 2 wk period to their final starch intake. In ruminant animals, the proliferation of *S*. *bovis* only occurs when animals are un-adapted to grain feeding or during the step-up feeding period [20, 41, 42]. In fact, research has demonstrated that once cattle are adapted to a high grain diet, the number of *S*. *bovis* decline to be similar to the viable number in forage-fed cattle [16, 43, 44, 45]. Previous research in ponies un-adapted to grain feeding observed that viable numbers of lactobacilli and GPC (reported as streptococci), rapidly increased following an abrupt incorporation of barley into a hay diet (50% rolled barley, 50% hay) [13]. These researchers also reported an increase in GPC and lactobacilli after ponies were adapted to the same diet for 14 d [24]. However, they did not report enumerations over the course of adaptation to the barley diet, and to our knowledge, the effect of grain adaptation on predominant amylolytic bacteria and *S*. *bovis* proliferation has not been studied in horses. Therefore, it could be speculated that the equine hindgut would respond to starch and adaptation in a similar manner as the rumen.

In an acutely acidic rumen, *S*. *bovis* proliferation is only transient, and viable numbers decline while lactobacilli increase, indicating a potential competitive relationship between these bacteria [16, 41, 43, 44, 45]. Similarly, in the current study, a strong negative correlation was identified between the viable number of lactobacilli and GPC (*Enterococcus* spp., *S*. *bovis/equinus*) and the viable number of lactobacilli and total amylolytic bacteria on S2 (r = -0.99 and r = -0.97, respectively), indicating a potential competitive relationship between these bacteria. Amylolytic bacterial competition could be explained by several different factors, including both plant-specific factors and bacteria-specific factors.

The plant-specific factors that could influence starch fermentation in the hindgut include starch chemistry, non-starch components, and endosperm structure. Corn, oats and wheat starches differ both in composition (proportion of amylose and amylopectin) and morphology [3, 4, 5, 6, 7]. It is also possible that a non-starch component of the grain could influence bacterial competition. For example, oats contain the soluble fiber, β-glucan. Although, research is limited on β-glucan fermentation in the equine hindgut, human research has suggested that oat β-glucan is completely fermented in the colon by *Bacteroides* spp., producing short chain fatty acids for host utilization and promoting normal microbiota stability [46]. Additionally, Snart and colleagues (2006) demonstrated that β-glucans could act as a prebiotic, promoting the growth of lactobacilli [47].

Bacteria-specific factors (e.g., substrate affinity, substrate preference, pH tolerance) could also influence competition among amylolytic bacteria [48]. Bacterial amylases can vary greatly in active site structure and substrate specificity and affinity, which could allow one bacterium to outcompete another for starch substrate [49, 50, 51]. A few studies have been done to look at the effect of substrate affinity on amylolytic bacteria competition [52, 53, 54]. In addition, McAllister and colleagues (1990) compared the capacity of 3 ruminal amylolytic bacteria (*S*. *bovis*, *Ruminobacter amylophilus* and *Butyrvibrio fibrosolvens*) to digest corn, wheat or barley whole grain flour [55]. Although the rates of starch digestion by *S*. *bovis* were similar regardless of grain type, several notable differences were observed with *R*. *amylophilus* and *B*. *fibrosolvens*. *R*. *amylophilus* preferentially colonized barley and had higher percentages of starch disappearance than when incubated with corn or wheat starch. In contrast, *B*. *fibrosolvens* digested similar amounts of barley and corn starch but little wheat starch. These observations support the concept that starch fermentation is influenced by both starch source and microorganism present to digest the starch. Additionally, many amylolytic bacteria are capable of producing bacteriocins and other antimicrobial molecules that could influence bacterial competition [36, 56, 57, 58, 59]. For example, *E*. *faecalis* and *S*. *bovis* produce compounds with antimicrobial activity against *Lactobacillus* spp. and each other [38, 56, 59].

Addition of starch to equine diets has often been linked to decreased fibrolytic activity. Thompson and coworkers (1984) found that fiber digestibility decreased in horses when 60% or more of the dietary forage was replaced with oats [60]. Replacing hay with barley resulted in decreased fiber digestibility as well as a decrease in cellulolytic bacteria in the large intestine of fistulated ponies [24, 61]. Similarly, Medina *et al*. (2002) replaced dehydrated alfalfa with barley and reported a decrease in cellulolytic bacteria in the cecum of fistulated horses [14]. In those studies, the increase in starch intake was confounded with a decrease in fiber intake. In our study, the starch sources were fed in addition to the hay and the amount of hay that was fed during the sampling period remained constant for all horses. Thus, the decrease in cellulolytic bacteria in horses fed LC, LW, HC and HO can be more confidently attributed to the addition of starch to the diet and not to a decrease in dietary fiber intake. Furthermore, in ruminants, pH has been reported to inhibit the metabolism and growth of fibrolytic bacteria [53]. It is possible that the decreased pH in the horses fed LC, LW and HC was a factor in the observed decrease in number of cellulolytic bacteria in those treatments.

It is important to consider that the starch concentrations fed in the current study were representative of feeding rates used for horses in many practical situations. Additionally, no horses displayed any outward symptoms of acidosis or related conditions over the course of the study. Potter *et al*. (1992) suggested that the upper limit of starch digestion in the equine small intestine was between 3.5 and 4 g starch/kg BW per feeding [9]. However, others have suggested that to limit starch bypass to the large intestine, starch intake should not exceed 2 g/kg BW per meal [10, 62]. In this study, changes to the fecal bacterial community occurred when horses were fed LW and LC. This latter result suggests that starch bypass to the large intestine occurs even at starch intakes below 2 g starch/kg BW per meal, at least when corn and wheat middlings were fed. The extent of starch digestion in the small intestine can be affected by processing [9, 10], so the amount of starch that will result in bypass to the large intestine is likely to vary with both starch source and processing. In this study, the wheat middlings were pelleted, the corn was coarsely cracked and oats had been cleaned but not otherwise processed. Grinding and pelleting the oats and the corn may have resulted in less bypass of starch and fewer effects on the microbial ecosystem.

More marked changes might have been noted if the addition of a high level of starch to the diet had been more abrupt. The current study employed a gradual introduction of starch to the feeding program as is commonly recommended [26]. Thus, the observed changes represent what can occur during the initial period when cereal grain starch is gradually introduced to horses consuming a forage-based diet. This study did not examine changes to the fecal bacteria that might occur after long-term adaptation of horses to a starch-containing diet. Additional research is warranted to evaluate potential change to long term adaptation.

Overall, this study revealed that starch source can affect the microbial ecology of the equine digestive tract as measured in freshly voided feces. The use of feces as a proxy for microbial changes in the equine large intestine has become common [15, 27, 29, 32, 63, 64, 65]. Feces collection is minimally invasive whereas the fistulation of horses or ponies in the cecum or colon is major surgery and the cannulae may not be tolerated well in the long term. Conclusions drawn from changes observed in feces should be viewed cautiously as they may not precisely reflect either the magnitude or the time-course of changes in the cecum or colon. However, it seems likely that if differences due to diet are observed in feces at the end of the digestive process, that those differences would have been present in the earlier sites of digestion as well.

## Conclusions

A number of previous studies demonstrated that adding a high-starch concentrate to a predominantly forage diet can alter the equine gastrointestinal microbial community. The current study also found that adding starch to the diet can alter the microbial community and it expanded those findings by examining differences due to starch source (corn, oats or wheat middlings). At equal starch intakes, corn produced more marked changes in the fecal microbial ecosystem than oats. Therefore, differences in composition and morphology of the starch granule, or in other components of the grains, could also affect amylolytic proliferation. A variety of predominant amylolytic bacteria were observed in horses fed hay and oats. However, the predominant amylolytic bacterium in corn and wheat fed horses was *E*. *faecalis*, which was present at much greater number (10,000-fold) in the horses on these diets. To develop a better understanding of the pathogenesis of several detrimental health conditions (*i*.*e*., colic, laminitis) that can be triggered by carbohydrate overload, as well as aid in the development of effective treatment strategies, further studies using higher levels of starch intake are needed.

## Supporting Information

S1 Table Equine amylolytic isolate closest phylogenetic relative (> 97% 16S gene sequence identity) and characterization. (DOCX)
