# Supplementary material for: Effect of Dietary Starch Source and Concentration on Equine Fecal Microbiota
Source: PLoS One. 2016 Apr 29;11(4):e0154037. doi: 10.1371/journal.pone.0154037 (PMC4851386; doi:10.1371/journal.pone.0154037)
Supplement: S1 Table — (DOCX) [file pone.0154037.s001.docx]

**S1 Table: Equine amylolytic isolate closest phylogenetic relative (> 97% 16S gene sequence identity) and characterization.**

|  | **S** | **Blk** | **16S ID** | **GenBank Accession Number** | **Substrate Utilization** | | | **Blood Hemolysis** |
| --- | --- | --- | --- | --- | --- | --- | --- | --- |
|  |  |  |  |  | **Glc** | **In** | **Esc** |  |
| **CON** | 0 | 1 | *Enterococcus faecalis* | KX073757 | + | + | + | None |
|  |  | 3 | *Enterococcus faecalis* | KX073774 | + | + | + | None |
|  |  | 4 | *Enterococcus faecalis* | KX073789 | + | + | + | None |
|  |  | 5 | *Enterococcus faecalis* | KX073804 | + | + | + | None |
|  | 1 | 3 | *Enterococcus faecalis* | KX073779 | + | + | + | None |
|  |  | 4 | *Enterococcus faecalis* | KX073795 | + | + | + | None |
|  |  | 5 | *Actinobacillus succinogenes* | KX073808 | + | + | + | None |
|  | 2 | 1 | *Enterococcus faecalis* | KX073768 | + | + | + | None |
|  |  | 3 | *Enterococcus faecalis* | KX073786 | + | + | + | None |
|  |  | 5 | *Enterococcus faecalis* | KX073812 | + | + | + | None |
| **HC** | 0 | 1 | *Enterococcus faecalis* | KX073752 | + | + | + | None |
|  |  | 3 | *Escherichia coli* | KX073769 | + | + | + | None |
|  |  | 4 | *Streptococcus macedonicus* | KX073787 | + | + | + | None |
|  |  | 5 | *Streptococcus bovis* | KX073799 | + | + | + | None |
|  | 1 | 1 | *Enterococcus faecalis* | KX073758 | + | + | + | None |
|  |  | 4 | *Enterococcus faecalis* | KX073790 | + | + | + | None |
|  |  | 5 | *Enterococcus avium* | KX073805 | + | + | - | None |
|  | 2 | 1 | *Enterococcus faecalis* | KX073763 | + | + | + | None |
|  |  | 3 | *Enterococcus faecalis* | KX073780 | + | + | + | None |
|  |  | 5 | *Enterococcus faecalis* | KX073809 | + | + | + | None |
| **HO** | 0 | 1 | *Streptococcus macedonicus* | KX073753 | + | + | + | None |
|  |  | 3 | *Enterococcus faecalis* | KX073770 | + | + | + | None |
|  |  | 5 | *Enterococcus faecalis* | KX073800 | + | + | + | None |
|  | 1 | 1 | *Streptococcus bovis* | KX073759 | + | + | + | None |
|  |  | 3 | *Enterococcus faecalis* | KX073775 | + | + | + | None |
|  |  | 4 | *Enterococcus faecalis* | KX073791 | + | + | + | None |
|  | 2 | 1 | *Lactococcus lactis* | KX073764 | + | + | - | None |
|  |  | 3 | *Enterococcus avium* | KX073781 | + | + | - | None |
|  |  | 3 | *Streptococcus bovis* | KX073782 | + | + | + | None |
|  |  | 5 | *Clostridium sordellii* | KX073810 | + | + | + | None |
| **LC** | 0 | 1 | *Lactococcus lactis* | KX073754 | + | + | - | None |
|  |  | 3 | *Enterococcus faecalis* | KX073771 | + | + | + | None |
|  |  | 5 | *Streptococcus macedonicus* | KX073801 | + | + | + | None |
|  | 1 | 1 | *Streptococcus bovis* | KX073760 | + | + | + | None |
|  |  | 3 | *Enterococcus faecalis* | KX073776 | + | + | + | None |
|  |  | 4 | *Enterococcus faecalis* | KX073792 | + | + | + | None |
|  |  | 5 | *Enterococcus avium* | KX073806 | + | + | - | None |
|  | 2 | 1 | *Enterococcus faecalis* | KX073765 | + | + | + | None |
|  |  | 3 | *Enterococcus faecalis* | KX073783 | + | + | + | None |
|  |  | 4 | *Enterococcus faecalis* | KX073796 | + | + | + | None |
| **LO** | 0 | 1 | *Streptococcus bovis* | KX073755 | + | + | + | None |
|  |  | 3 | *Enterococcus faecalis* | KX073772 | + | + | + | None |
|  |  | 5 | *Enterococcus faecalis* | KX073802 | + | + | + | None |
|  | 1 | 1 | *Enterococcus faecalis* | KX073761 | + | + | + | None |
|  |  | 3 | *Enterococcus faecalis* | KX073777 | + | + | + | None |
|  |  | 4 | *Lactococcus lactis* | KX073793 | + | + | - | None |
|  |  | 5 | *Streptococcus criceti* | KX073807 | + | + | + | None |
|  | 2 | 1 | *Enterococcus avium* | KX073766 | + | + | - | None |
|  |  | 3 | *Enterococcus faecalis* | KX073784 | + | + | + | None |
|  |  | 4 | *Streptococcus bovis* | KX073797 | + | + | + | Alpha |
| **LW** | 0 | 1 | *Actinobacillus succinogenes* | KX073756 | + | + | + | None |
|  |  | 3 | *Enterococcus faecalis* | KX073773 | + | + | + | None |
|  |  | 4 | *Streptococcus macedonicus* | KX073788 | + | + | + | None |
|  |  | 5 | *Streptococcus bovis* | KX073803 | + | + | + | None |
|  | 1 | 1 | *Enterococcus faecalis* | KX073762 | + | + | + | None |
|  |  | 3 | *Enterococcus faecalis* | KX073778 | + | + | + | None |
|  |  | 4 | *Enterococcus faecalis* | KX073794 | + | + | + | None |
|  | 2 | 1 | *Enterococcus faecalis* | KX073767 | + | + | + | None |
|  |  | 3 | *Enterococcus faecalis* | KX073785 | + | + | + | None |
|  |  | 4 | *Enterococcus faecalis* | KX073798 | + | + | + | None |
|  |  | 5 | *Enterococcus faecalis* | KX073811 | + | + | + | None |

CON (hay only), HC (high corn), HO (high oats), LC (low corn), LO (low oats), LW (low wheat); high = 2 g starch/kg BW, low = 1 g starch/kg BW

Day S0 (hay only), Day S1 (d 6; 50% final starch intake), Day S2 (d 13; 100% final starch intake). Substrate utilization: Glucose (Glc), inulin (In) or esculin (Esc).
